# A Rare Case of Plasmacytoma Presenting as Pulmonary Mass

**DOI:** 10.1002/rcr2.70168

**Published:** 2025-03-25

**Authors:** Qiang‐zhong Pi, Hu Luo

**Affiliations:** ^1^ Department of Respiratory and Critical Care Medicine The First Affiliated Hospital of Army Medical University (Southwest Hospital) Chongqing China

**Keywords:** extramedullary plasmacytoma, plasmacytoma, pulmonary mass

## Abstract

Plasmacytoma can manifest as a solitary pulmonary mass, which can be accurately diagnosed through a combination of Bone scan, serum protein electrophoresis, computed tomography (CT)‐guided biopsy and bone marrow biopsy. Notably, this case diagnosed with multiple myeloma (MM) with extramedullary plasmacytoma (EMP) demonstrated an exceptional treatment response to radiotherapy and proteasome inhibitor–based chemotherapy.

## Graphical abstract

We report a patient diagnosed with multiple myeloma (MM) with extramedullary plasmacytoma (EMP) who demonstrated an exceptional treatment response to radiotherapy and proteasome inhibitor–based chemotherapy.

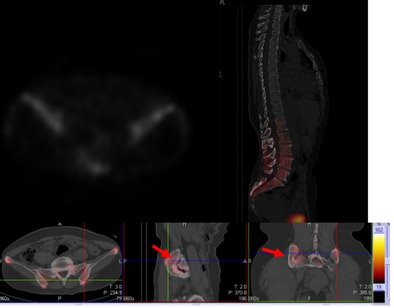

## 1 Clinical Image

**FIGURE 1:**
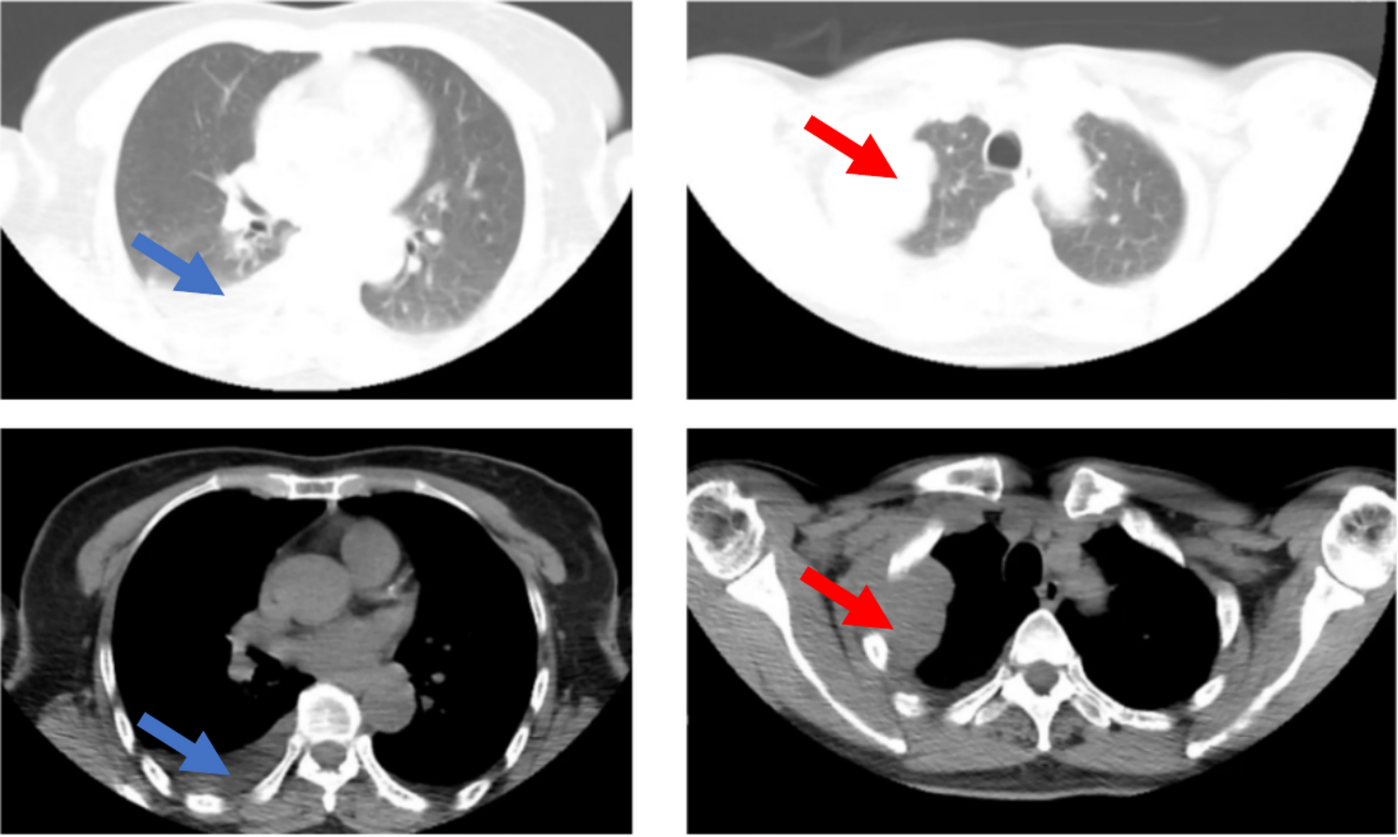
Non‐contrast chest CT scan of chest shows large right‐sided mass (red arrow) with osseous erosion of ribs 2–4 and intercostal muscle infiltration on May 5, 2024. This designation aligns with the imaging protocol chosen to avoid nephrotoxic contrast media in a patient with borderline low albumin (31.0 g/L). The well‐defined solid mass (oval shape with lobulated margins) is in the right upper lobe (segmental bronchus 3) with homogeneous attenuation (35–40 HU), mild pleural thickening adjacent to the mass and small right pleural effusions (blue arrow). CT, computed tomography.

**FIGURE 2:**
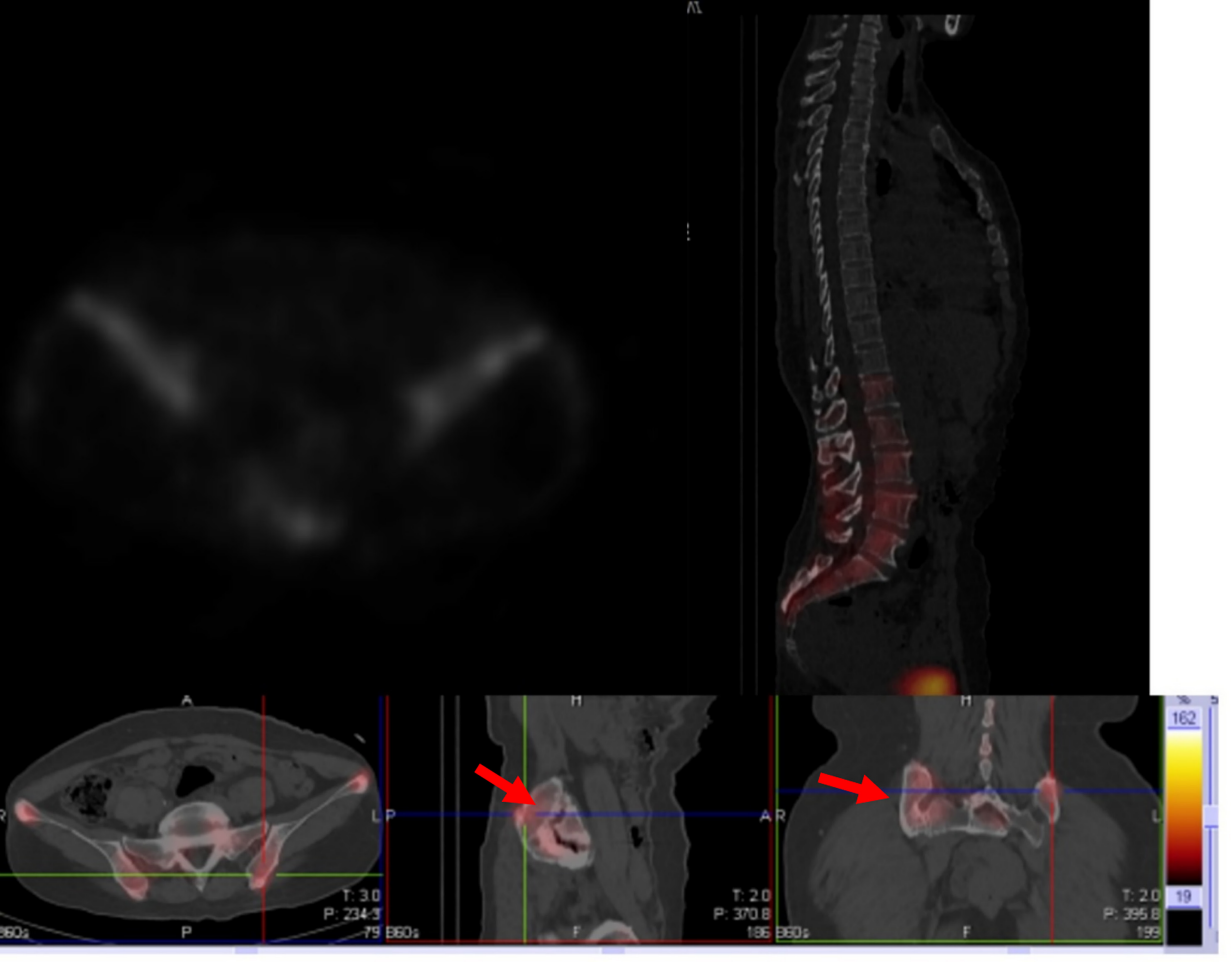
Bone scan showed uneven radioactive distribution in multiple bones throughout the body. Notably, abnormal metabolic activity is observed in both humeri (as red arrow). Considering the aforementioned findings, tumorous lesions are suggested, with a suspicion of hematologic malignancy.

A 56‐year‐old asymptomatic female was found to have a 40 × 36 mm^2^ right upper lobe mass on routine chest CT in May 2024 (Figure 1). Physical examination revealed diminished right‐sided vocal fremitus in the upper lung field. There was monoclonal IgG‐λ gammopathy (IgG 45 g/L, normal 7–16 g/L) with elevated λ light chains (520 mg/L, normal 50–260 mg/L) and a reversed κ/λ ratio (0.16, normal 1.35–2.65) and anaemia (Hb 82 g/L, normal 115–150 g/L for females) without abnormal renal function. A bone scan found multifocal hypermetabolic lesions in bilateral humeri and iliac bone (Figure 2E). Bone marrow examination showed 17% plasma cells with low intracellular iron. Rib erosion and extrapulmonary extension were seen on CT and confirmed by biopsy as plasmacytoma.

**FIGURE 3:**
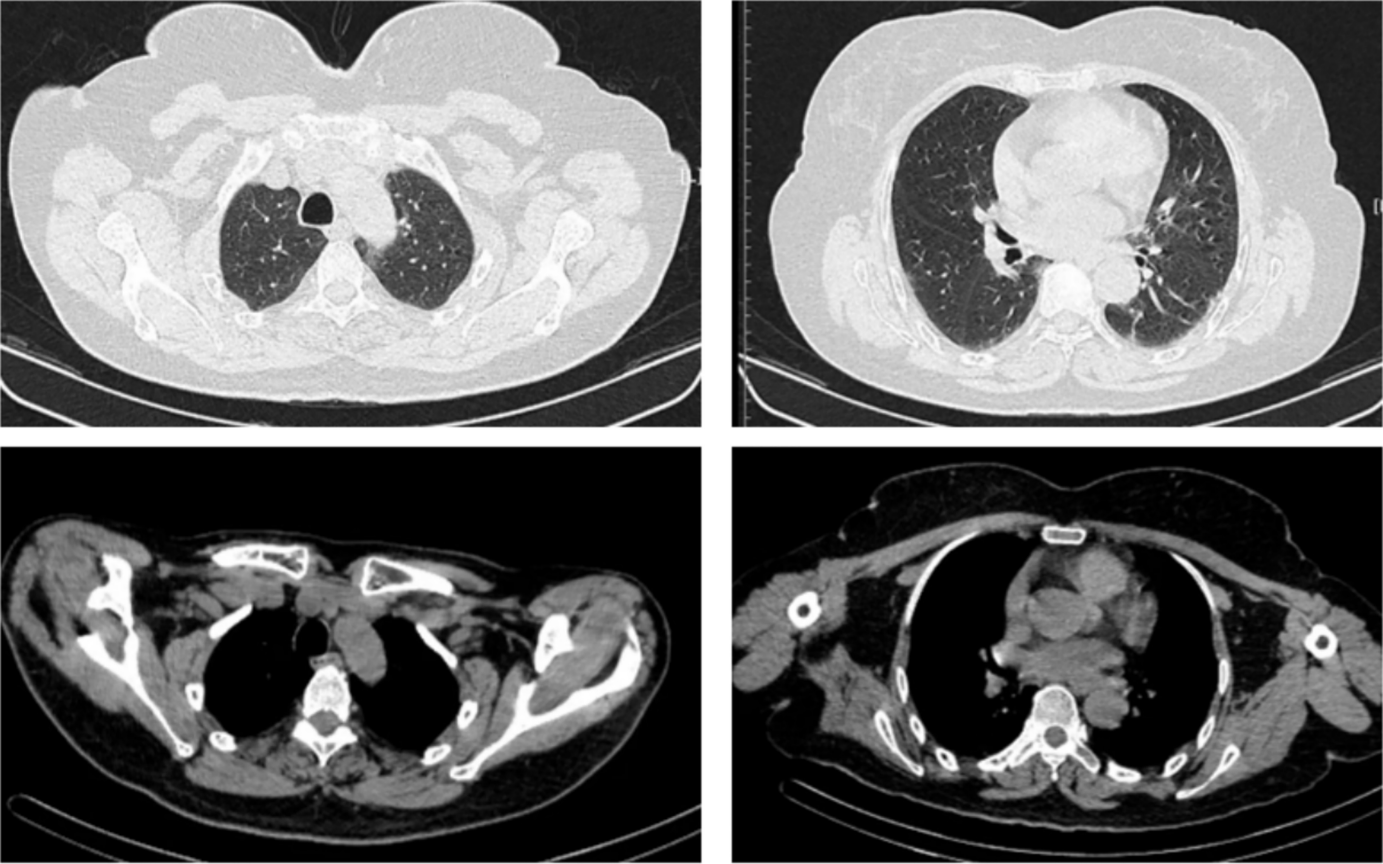
The non‐contrast chest CT scan on September 9, 2024, demonstrated complete resolution of the pulmonary mass following 4 cycles of bortezomib, dexamethasone, and thalidomide therapy.

The patient received bortezomib, dexamethasone and thalidomide. After 4 cycles, the mass resolved (Figure 3) and Hb normalised to 128 g/L. Serum protein electrophoresis and M‐protein became negative, achieving stringent complete remission. EMPs occur in 7%–18% of MM patients [1] but pulmonary involvement is rare. While thalidomide is ineffective for EMPs, proteasome inhibitors (e.g., bortezomib) combined with immunomodulatory drugs have shown prolonged overall survival in relapsed EMPs [2]. This case highlights the efficacy of such regimens in achieving deep responses even in challenging anatomical locations.

## Author Contributions

**Qiang‐zhong Pi:** conception and design of the study, collected the data, and drafted the initial manuscript. **Hu Luo:** refine the manuscript for clarity and coherence.

## Ethics Statement

The authors declare that appropriate written informed consent was obtained for the publication of this manuscript and accompanying images.

## Conflicts of Interest

The authors declare no conflicts of interest.

## Data Availability

Data available on request due to privacy/ethical restrictions.
